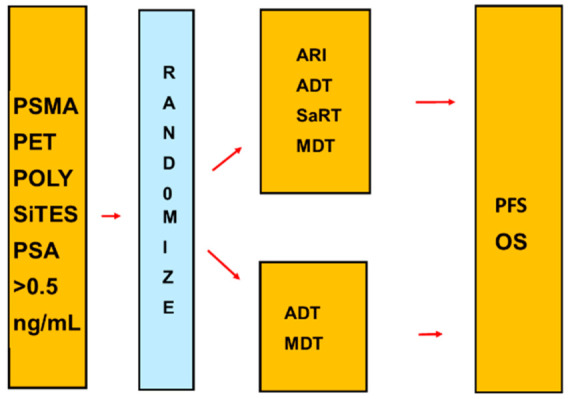# Correction: von Eyben et al. A Risk Model for Patients with PSA-Only Recurrence (Biochemical Recurrence) Based on PSA and PSMA PET/CT: An Individual Patient Data Meta-Analysis. *Cancers* 2022, *14*, 5461

**DOI:** 10.3390/cancers15041035

**Published:** 2023-02-07

**Authors:** Rie von Eyben, Daniel S. Kapp, Manuela Andrea Hoffmann, Cigdem Soydal, Christian Uprimny, Irene Virgolini, Murat Tuncel, Mathieu Gauthé, Finn E. von Eyben

**Affiliations:** 1 Cytel Incorporated, 1050 Winter St, Waltam, MA 02452, USA; 2 Department of Radiation Oncology, Stanford University School of Medicine, Palo Alto, CA 94305, USA; 3 Department of Occupational Health & Safety, Federal Ministry of Defense, 53123 Bonn, Germany; 4 Department of Nuclear Medicine, University Medical Center, Johannes Gutenberg University in Mainz, 55101 Mainz, Germany; 5 Department of Nuclear Medicine, University of Ankara, Ankara 06100, Turkey; 6 Department of Nuclear Medicine, University Hospital in Innsbruck, 6020 Innsbruck, Austria; 7 Department of Nuclear Medicine, Hacettepe University, Ankara 06230, Turkey; 8 Department of Nuclear Medicine, Incept, Institute Holland, 38100 Grenoble, France; 9 Center of Tobacco Control Research, 5320 Odense, Denmark

## Error in Figure

In the original publication [1], there was a mistake in Figure 6. The lower line that shows the control group treatment is ADT + SDaRT + MDT; however, it should only include ADT + MDT. The corrected Figure 6 appears below. The authors state that the scientific conclusions are unaffected. This correction was approved by the Academic Editor. The original publication has also been updated.

## Corrected Figure 6